# Impact of Pre-Procedure Interventions on No-Show Rate in Pediatric Endoscopy

**DOI:** 10.3390/children2010089

**Published:** 2015-03-17

**Authors:** Jyoti Mani, Linda Franklin, Harpreet Pall

**Affiliations:** 1 Section of Gastroenterology, Hepatology, and Nutrition, St. Christopher’s Hospital for Children, Nelson Pavilion 3635 North Front Street, Philadelphia, PA 19134, USA; E-Mails: jyotim1@gmail.com (J.M.); linda.franklin@tenethealth.com (L.F.); 2 Department of Pediatrics, Drexel University College of Medicine, 2900 West Queen Lane, Philadelphia, PA 19129, USA

**Keywords:** endoscopy, pediatrics, quality improvement

## Abstract

Pediatric endoscopy has evolved into an indispensable tool in the diagnosis and management of gastrointestinal diseases in children. However, there is limited literature focusing on quality improvement initiatives in pediatric endoscopy. The primary goal of this project was to reduce the no-show rate in the pediatric endoscopy unit. Also, we aimed to improve patient and family satisfaction with the procedure by identifying opportunities for improvement. A checklist was designed based on the potential causes of no-show. The endoscopy nurse coordinator reviewed the checklist when scheduling the procedure to identify patients at high risk for non-compliance. Once a risk factor was identified, appropriate actions were taken. She also made a pre-procedure phone call as a reminder and to address any of these risks for non-compliance if present. A patient satisfaction survey was used to identify potential areas for improvement. The no-show rate decreased from an average of 7% in the pre-intervention phase to 2% in the post-intervention phase (*p* = 0.009). 91% of the patients/family recorded an overall satisfaction of 4 or 5 on a scale of 1–5 (5 being best). Quality improvement strategies decreased the no-show rate in the pediatric endoscopy unit. A patient satisfaction survey helped in identifying areas for improvement.

## 1. Introduction

Pediatric endoscopy has evolved into an indispensable tool in the diagnosis and management of gastrointestinal diseases in children [1,2]. However, there is limited literature focusing on quality improvement initiatives in pediatric endoscopy [2]. An essential aspect of health care is to maintain optimal utilization of time and resources by ensuring patients keep scheduled appointments [3,4,5]. Patients who miss appointments disrupt the schedule/workflow of the endoscopy unit and also prevent other patients from receiving care in a timely manner. It is necessary to explore underlying risk factors for the phenomenon of no-show so as to optimize patient care. This would help to identify patients at high risk of non-compliance, enabling intervention at an early stage with specific targeted efforts to avert a no-show visit.

An important determinant of care is the level of patient and family satisfaction after an endoscopic procedure [6]. Analyzing quality of care from the perspective of the patient and family provides a better understanding of present services offered and also helps to identify areas for potential improvement [6].

St. Christopher’s Hospital for Children is a tertiary care academic pediatric medical center in Philadelphia and serves a diverse patient population with a significant proportion of patients from a low socioeconomic background. The Section of Pediatric Gastroenterology consists of seven gastroenterologists. As the program has grown, the volume of endoscopic procedures has substantially increased. Patient no-show creates a bottle-neck effect limiting the number of procedures that can be performed. Missed appointments result in significant loss of time and resources compromising the efficiency and quality of care [3,4]. Before the study period, our department did not have specific strategies to address this complex problem. A quality improvement (QI) team was formed at St. Christopher’s Hospital for Children in January 2013 with the primary goals of reducing the no-show rate in the endoscopy unit and improving patient and family satisfaction.

## 2. Materials and Methods

This study was performed by the endoscopy QI team at St. Christopher’s Hospital for Children, a tertiary care children’s hospital serving as the pediatric teaching hospital for Drexel University College of Medicine. The QI team consisted of gastroenterologists, endoscopy nurse coordinator, medical social worker, child life specialist and QI coordinator. There was no conflict of interest among the team members and the study was initiated as part of our commitment to provide high quality patient-centered care. The study was exempted from IRB review as it was deemed a quality improvement initiative.

### 2.1. Scheduling Process for Pediatric Endoscopy

After the patient was identified as needing an endoscopic procedure, the physician reviewed the procedure with family including its risks, benefits and alternatives. The patient was then directed to the endoscopy nurse coordinator who helped with scheduling the procedure and explaining any specific preparation (*i.e.* bowel preparation prior to colonoscopy). Depending on risk factors, some patients needed pre-operative anesthetic assessments. This was determined by guidelines from our anesthesia department. The endoscopy unit is used five days a week by seven gastroenterologists, with block time assigned to each gastroenterologist. Our study was geared towards patients who were scheduled for outpatient endoscopic procedures such as esophago-gastro-duodenoscopy and colonoscopy.

**Figure 1:**
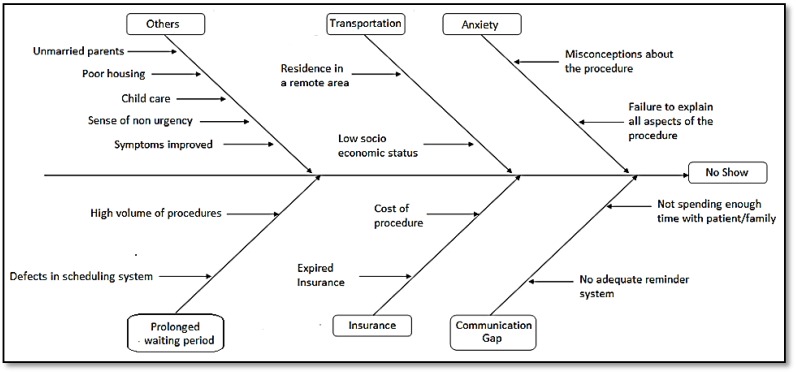
Cause and effect (fishbone) diagram of reasons for no-show in pediatric endoscopy.

A no-show was defined as missing the scheduled procedure without calling in to cancel. The no-show rate for each month was calculated as the ratio of number of no-shows to total procedures scheduled for the month. The initial objective was to measure the baseline incidence of no-show in the endoscopy unit over six months. We reviewed existing literature and examined the no-show history of our endoscopy unit to identify potential risk factors and possible causes [3,4,5,7,8]. The causes were represented on a cause and effect fishbone/Ishikawa diagram (Figure 1). In an ideal state these potential risks/causative factors would be addressed at an early stage with appropriate interventions. Based on the conclusions inferred from the above analyses, the team designed a multi-method intervention to decrease the no-show rate in our endoscopy unit and improve patient and family satisfaction (Table 1). Primary process interventions included running Plan-Do-Study-Act cycles, using a comprehensive checklist to identify patients at risk and routinely calling all patients’ families 48–72 h prior to the procedure.

### 2.2. The No-Show Checklist and Pre-Procedure Phone Call

A checklist comprising potential causes for no-show was designed to identify patients at risk (Table 2). The major causes of no-show based on existing literature and the no-show history of our endoscopy unit were utilized in designing the check-list. The endoscopy nurse coordinator reviewed the checklist when scheduling the procedure in all patients to identify those who may be at a higher risk for non-compliance. Once a risk factor was identified, appropriate interventions were undertaken. A medical social worker was utilized for many of the interventions. The endoscopy nurse coordinator also made a pre-procedure phone call to all scheduled patients approximately 48–72 h before the procedure as a reminder and also to address any questions or concerns they had at that time. At the time of the phone call, she again reviewed the checklist to ensure risk factors for no-show were not present.

**Table 1:** Summary of interventions that were implemented to reduce the no-show rate and improve patient satisfaction in our pediatric endoscopy unit.

Stage of Intervention	Action
Endoscopy nurse schedules date for procedure	Review of check list assessing all causes for no-show and appropriate action if needed
Endoscopy nurse makes phone call 48–72 h before procedure	Phone call serves as reminder and also provides family/patient an opportunity to address concerns before procedure
Patient answers questionnaire after procedure in recovery room	Appropriate action taken depending on identified gaps in patient experience

**Table 2:** No-show risk checklist. The checklist was used by the endoscopy nurse coordinator when scheduling the procedure to identify patients at high risk for no-show.

#	Check List Questions	Response		Steps
1	Does the patient have any insurance related issues that might result in cancellation of the scheduled procedure?	□ Yes	□ No	If Yes direct the patient to the Social Worker
2	Does the patient have any transportation issues that might prevent him/her from arriving for the procedure?	□ Yes	□ No	If Yes direct the patient to the Social Worker
3	Does the patient have a previous history of no-show in the Endoscopy Unit?	□ Yes	□ No	If Yes direct the patient to the Social Worker
4	Did you inform the family about the role played by the Child Life Specialist/Social Worker and how they could contact them if they feel it is necessary?	□ Yes	□ No	If No let the patient know of their availability for immediate assistance
5	Does the patient have a high level of anxiety needing additional psychological preparation?	□ Yes	□ No	If Yes direct the patient to the Social Worker
6	Does the patient have a clear understanding of the procedure and preparation?	□ Yes	□ No	If No spend more time with the patient/family to explain the procedure and clear any misconceptions

For example, if a patient resided in a remote area with poor access to public transport he/she was considered at high risk for no-show. The patient was referred to our social worker who worked with the family to facilitate scheduling the procedure. The social worker also made transportation arrangements if necessary. Similarly, a patient/family with high anxiety was referred to the social worker for additional counseling and preparation for the procedure, including the use of child friendly books explaining the procedure. Patients with previous history of no-show to the endoscopy unit were considered at higher risk for no-show. In these patients the social worker further ensured the family understood the significance of the procedure and was well prepared.

### 2.3. Questionnaire survey

A questionnaire survey covering various aspects of care was used during the intervention period to identify potential areas needing improvement (Supplemental Table 1). Factors contributing to patient satisfaction were identified by literature review and incorporated to design the questionnaire. The Global Rating Scale which is used in adults to assess endoscopy performance was also reviewed when designing the questionnaire [9]. The survey was administered to the parents after the procedure when waiting in the recovery room.

### 2.4. Outcomes

The no-show rate was monitored during the study period before and after the intervention. The responses to the questionnaire survey were analyzed to identify gaps in the patient-experience.

### 2.5. Data Analysis

The difference in the mean no-show rate in the pre-intervention and post-intervention phase was calculated using the Chi square test. A *p* value < 0.05 was considered significant. The data was analyzed using the SPSS (version 19.0, IBM, New York, NY, USA). The outcome measures were analyzed by using statistical process control charts. Sample size and power were not calculated as this was a pilot study.

## 3. Results

**Figure 2:**
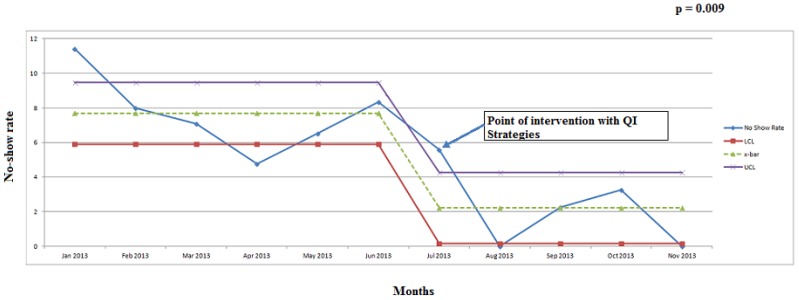
Statistical process control chart representing no-show rate. Statistical process control chart representing no-show rate in pediatric endoscopy unit from January–November 2013. ucl—upper control limit ($\bar{x}$ + 3 SD); lcl—lower control limit ($\bar{x}$ − 3 SD); $\bar{x}$—mean no-show rate; QI—quality improvement; SD—standard deviation.

The no-show rate decreased from an average of 7% (22 out of 298 scheduled procedures) in the pre-intervention phase (January–June 2013) to 2% (5 out of 223 scheduled procedures) in the post-intervention phase (July–November 2013), *p* = 0.009 (Figure 2 and Table 3). We analyzed the survey results from 33 patients who underwent a procedure from September–October 2013. 91% of the patients/family recorded an overall satisfaction of 4 or 5 on a scale of 1–5 (5 being best).

Summary of the number of procedures that were performed/scheduled and no-shows during the study period; January–November 2013.

**Table 3:** Monthly number of procedures scheduled/performed and no-shows.

Month	January	February	March	April	May	June	July	August	September	October	November
Procedures scheduled	35	50	56	63	46	48	36	39	44	61	43
Procedures performed	31	46	52	60	43	44	34	39	43	59	43
No-shows	4	4	4	3	3	4	2	0	1	2	0
No-show rate (%)	11.40%	8.00%	7.10%	4.76%	6.52%	8.33%	5.55%	0.00%	2.27%	3.27%	0.00%

All families who took the survey (n = 33) responded that the procedure was clearly explained to them including risks, benefits, alternatives and the need for sedation. Also, all of them felt they received enough information to be well prepared for the procedure including an opportunity to clarify any of their concerns. 33% of families were not getting a supplemental booklet further explaining the procedure. 76% of families reported that they were given a choice of dates when scheduling the procedure. 97% of families felt that the procedure was scheduled in a timely manner after being seen in the clinic. On a scale of 1–5 (1 being very relaxed and 5 being very anxious), 51% of the parents rated anxiety of their child for the procedure as 1 and 15% rated it as 4 or 5. 71% of families stated that the procedure was done in a timely manner after arrival in the endoscopy unit on the day of procedure. Once it was identified that some patients were not receiving a booklet further explaining the procedure, adequate steps were taken to ensure that there were enough copies in the main hospital and satellite clinics. 

## 4. Discussion

The field of pediatric gastrointestinal endoscopy has continued to evolve over the past decade [1,2]. It is critical to enhance efficiency and deliver high quality patient-centered care through QI initiatives [2]. The QI study carried out in our endoscopy unit resulted in a significant decrease in no-show rate with implementation of a comprehensive no-show checklist and pre-procedure phone call. A patient satisfaction survey helped to assess quality of services and to identify possible areas for improvement.

Patient no-show is a key barrier that needs to be addressed as it can cause unnecessary wastage of time, resources and manpower [3,4,5]. Missed appointments negatively affect the continuity and quality of care for both patients who no-show and for others who could have been scheduled in those appointment slots [3,4,5].

To date, there is limited data on quality improvement studies focusing on no-show rate in pediatric endoscopy. Most studies in the literature have focused on no-show rate in adult endoscopy units, operating rooms and outpatient clinics [3,4,5,7,8]. In 1996, a study was carried out by McClure *et al.* to identify the characteristics of children who no-show for appointments [5]. Over six months, 359 children having an appointment at a general clinic were studied using a questionnaire given to parents (74% response rate) and by inspection of case notes. Based on their first appointment in the study period, children were divided into “attenders” (n = 262) and “non-attenders” (n = 97). Non-attenders were significantly more likely to have one or more of the following characteristics: lower social class, poorer housing, unmarried parent(s) (56% *vs.* 33%), longer journey to clinic (35 *vs.* 27.6 minutes), more appointments per year (4.2 *vs.* 3.3), poorer past attendance record, and received their appointment by mail (76% *vs.* 44%). Surprisingly parents of non-attenders rated their children to have a significantly more severe illness than those who attended. These results suggest that attendance is primarily determined by social and logistical factors as well as appointment details, rather than illness severity.

Transportation issues, anxiety about the procedure, insurance related issues, lack of proper understanding, sense of non-urgency, misconceptions about the procedure and absence of an adequate reminder system are factors related to no-show [3,4,5,7,8]. Double booking strategies, reducing waiting time for appointment, patient reminders by telephone or email, patient education, and imposing a no-show fee are described methods to increase patient attendance [10,11,12,13].

In the current study, we clustered causes of no-show compiled from a literature review and team meetings [3,4,5,7,8]. A checklist was designed based on these potential causes to identify patients at risk. Our endoscopy nurse coordinator reviewed the checklist when scheduling the procedure and took appropriate actions in patients at higher risk for no-show. An aligned approach with our medical social worker was critical in this process. Also, the endoscopy nurse coordinator made a pre-procedure phone call 48–72 h before the procedure as a reminder and to again address any of these risks for noncompliance. The utility of a checklist as an effective QI tool is well documented in the literature. In 2013, Kwok *et al*. demonstrated the effectiveness of the World Health Organization Surgical Safety checklist in reducing surgical hazards and complications [14]. Also, telephone calls and other patient reminder systems have been found effective in reducing patient no-show in several previous studies [10,11,12,13]. The average wait time from clinic visit to procedure date during the study period was 2–3 weeks, which did not significantly change in the post-intervention phase. We did not do a cost analysis during the study period. However, the decreased no-show rate resulted in improved efficiency of the endoscopy unit and helped increase volume and revenue. 

We believe that implementation of this comprehensive checklist and pre-procedure phone call played a substantial role in bringing a significant decrease to our no-show rate. Our study demonstrates how quality improvement tools could be effectively utilized to systematically analyze the potential causes of missed appointments and formulate an action plan. To our knowledge, this is the first study demonstrating effectiveness of QI interventions in reducing no-show rate in a pediatric endoscopy unit.

Patient satisfaction surveys are key components of quality assurance projects and can help design novel ways to deliver patient-centered care [6,15]. Although there are valid tools such as the Global Rating Scale to assess patient satisfaction after endoscopy in adults, similar tools are not available in children [9]. Although families responded that they were receiving enough information, our survey results showed that some patients were not receiving a supplemental booklet further explaining the procedure. Once this issue was identified, adequate steps were taken to ensure that there were enough copies of the booklet in the main hospital and satellite clinics. The questionnaire survey in this study was an effective tool in assessing patient satisfaction and identifying areas for further improvement. Our next steps include modifying our scheduling system so as to give patients greater flexibility in scheduling a date, reducing anxiety of the child before the procedure and continuing to improve the efficiency of our endoscopy unit.

Although the results were statistically significant, a limitation of our study was its relatively short duration. Also, conducting the patient satisfaction survey in the pre-intervention phase and collecting the survey results from more patients would have provided better insight. Future studies should consider a larger sample and longer observation period. There may be other confounding variables (weather conditions, flu season, school vacation, *etc.*) playing a contributing role in influencing the no-show rate. Also, a review in person with the family to compile the reason for no-show may potentially generate a better understanding of the factors. We plan to continue monitoring our no-show rate and sustain the improvement by incorporating these interventions into the system.

## 5. Conclusions

QI tools can be effectively utilized to improve the efficiency of pediatric endoscopy units. Our QI initiative has demonstrated a significant decrease in the no-show rate with implementation of a comprehensive no-show checklist and pre-procedure phone call. A satisfaction survey is a useful tool to assess quality of services and to identify possible areas for improvement. We believe our QI efforts can encourage other endoscopy units to design similar studies assessing efficiency and to formulate innovative ways to enhance the quality of the system.

## Supplementary Files

Supplementary File 1
