# Supplementary material for: Impact of Pre-Procedure Interventions on No-Show Rate in Pediatric Endoscopy
Source: Children (Basel). 2015 Mar 17;2(1):89–97. doi: 10.3390/children2010089 (PMC4928745; doi:10.3390/children2010089)
Supplement: Supplementary File 1 [file children-02-00089-s001.docx]

Supplementary Materials

**Table S1.** Patient satisfaction survey—Pediatric GI Endoscopy Unit.

**Pediatrics Gastro Intestinal Endoscopy Unit St. Christopher's Hospital for Children, Philadelphia, PA**

**Before the Procedure**

The following questions are intended to rate your experience before the procedure.

**Patient Name**

**Age**

**Sex**

Male Female

**Date of Procedure**

**1) Which procedure did you have?**

EGD (Esophago-gastro-duodenoscopy)/Upper GI Endoscopy

Colonoscopy (Examination of Entire Colon)

EGD and Colonoscopy

EUS (Ultrasound Examination of Stomach)

Capsule Endoscopy

DBE (Bouble Ballon Enteroscopy)

**2) How long did you have to wait for an appointment?**

Less than a week

One to Two weeks

Two to Four weeks

More than a month

**3) In your opinion was the procedure done in a timely manner after being seen in the clinic?**

Yes No

**4) Were you offered a choice of dates for the procedure to be done?**

Yes No

**5-a) Did you get a pamphlet/booklet explaining what the procedure involved?**

Yes No

**5-b) If you received a pamphlet/booklet did it explain the procedure in a clear way?**

Yes No I didn't get any

**6) Did the Doctor explain clearly why exactly the procedure was being performed?**

Yes No

**Table S1.** *Cont.*

**7) Was the procedure explained clearly to you including the risks, benefits and alternate options?**

Yes No

**8) Did you understand all aspects of having a sedative injection for the procedure?**

Yes No

**9) Did the doctor/nurse address all of your concerns?**

Yes No

**10) Did you get an opportunity to ask any questions that you may have had?**

Yes No

**11) Did you feel that you received enough information to prepare yourself and your child for the procedure?**

Yes No

**12) Do you think that your child was prepared enough for the procedure?**

Yes No

**13) Were you informed about the role played by our Social Worker and how she could be easily contacted to address any of your concerns?**

Yes No

**14-a) Did you get an opportunity to talk to our Medical Social Worker?**

Yes No

**15) If you had an opportunity to talk to our social worker, did it help your child to be better prepared on the day of the procedure?**

Yes No

Bottom of Form

Top of Form

**The Procedure**

The following questions are intended to rate your experience during the day of the procedure.

**16) Was the procedure done in a timely manner after arrival in the endoscopy suite?**

Yes No

**17) Do you think that the endoscopic suite environment is child friendly?**

Yes No

**18) How would you rate the anxiety of your child in regards to undergoing the procedure?**

|  | 1 | 2 | 3 | 4 | 5 |  |
| --- | --- | --- | --- | --- | --- | --- |
| Very Relaxed |  |  |  |  |  | Very Anxious |

**Table S1.** *Cont.*

**19) Were the Nurses/Doctors doing the procedure courteous and considerate?**

Yes No

**Aftercare**

The following questions are intended to rate your experience after the procedure.

**20) Were you given written and verbal instructions on the adverse effects that may occur and the necessary steps to follow in such a situation?**

Yes No

**21) Were you given a telephone number with 24 hour coverage that would be available in the event of an emergency?**

Yes No

**22) In your opinion were you discharged from the endoscopy unit in a timely manner?**

Yes No

**23) Did you feel that the information you were given regarding follow up was clear and detailed enough?**

Yes No

**Overall**

The following questions are intended to rate your overall experience.

**24) Overall, How satisfied are you with the services that were offered in regards to the endoscopy procedure?**

|  | 1 | 2 | 3 | 4 | 5 |  |
| --- | --- | --- | --- | --- | --- | --- |
| Very Dissatisfied |  |  |  |  |  | Very Satisfied |

**25) Please comment on how we could improve the services that we offer in regards to the procedure.**
